# Predictive features of central lymph node metastasis in papillary thyroid microcarcinoma: Roles of active surveillance in over-treatment

**DOI:** 10.3389/fmed.2022.906648

**Published:** 2022-09-26

**Authors:** Bo Han, Sen Hao, Jixiang Wu, Jugao Fang, Zhengxue Han

**Affiliations:** ^1^Department of General Surgery, Beijing Tongren Hospital, Capital Medical University, Beijing, China; ^2^Department of Otolaryngology Head and Neck Surgery, Beijing Tongren Hospital, Capital Medical University, Beijing, China; ^3^Department of Head and Neck Surgery, Baotou Cancer Hospital, Baotou, China; ^4^Department of Oral and Maxillofacial-Head and Neck Oncology, Beijing Stomatological Hospital, Capital Medical University, Beijing, China

**Keywords:** active surveillance, cervical lymph node metastasis, central lymph node dissections, occult lymph node metastasis, papillary thyroid microcarcinoma

## Abstract

**Background:**

Low-risk papillary thyroid microcarcinoma (PTMC) without clinically evident lymph nodes, extrathyroidal expansions, and distant metastases may be candidates for active monitoring.

**Objective:**

The purpose of this research is to identify risk factors for papillary thyroid microcarcinoma (PTMC) metastasis to central cervical lymph nodes (CLNM) and to discuss the viability of an active surveillance strategy to minimize unnecessary therapy for patients.

**Methods:**

This single-center retrospective study was conducted on the data and medical records of the patients who were diagnosed with PTMC and underwent surgery at the Baotou Cancer Hospital, China, between January 1, 2018, and December 31, 2019. Both lobectomy and complete thyroid resections were performed, and central lymph node dissections (CLND) were used in all patients. Comparisons and analyses were conducted on the preoperative ultrasound (US) characteristics, the post-operation pathological results, and lymph node metastasis.

**Results:**

We analyzed 172 patients with PTMC with average age 48.32 ± 10.59 years old, with 31 males and 142 females. US testing showed 74 (43.0%) patients had suspicious lymph nodes; 31 (41.9%) had capsular invasion and 52 (30.2%) patients were confirmed to have CLNM. Based on logistic regression analysis, central lymph node metastasis was shown to be more common in individuals with PTMC who were older than 45 years old, male, and had tumors that lacked micro-calcification on US imaging. Postoperative pathology assessments suggested that 58 cases (33.7%) were more suitable candidates for active surveillance cohorts.

**Conclusions:**

While active surveillance might benefit many PTMC patients, treatments for the patients should also encompass occult lymph node metastasis, especially in patients with over 45 years old, male, tumor without micro-calcification in the US imaging. Furthermore, the prediction of lymph nodes in the central cervical *via* the preoperative US and the PTMC risk stratification accuracy need to be improved. Our findings showed about 30% of the patients with PTMC had no active surveillance high-risk factors but required surgical treatment. Fear of cancer in the PTMC patients, although informed of the details, is still the main reason for choosing surgical treatment over active surveillance.

## Introduction

Thyroid cancer prevalence has increased substantially over the last several decades, but mortality from the disease have stayed essentially stable (1, 2). About half of this increase may be attributable to better detection and diagnosis of slow-growing, non-cancerous tumors called intrathyroidal papillary thyroid microcarcinoma (PTMC) (3). The current definition of PTMC is papillary thyroid cancer (PTC) with an MTD ≤ 10 mm. Recent studies have shown that if left untreated, the vast majority of malignant cancers would not be fatal (4, 5). Thus, there is a risk of overdiagnosis if thyroid cancer is diagnosed only on the basis of the presence of these tiny tumors (6, 7). Accurate diagnostics made with modern ultrasound (US) and fine needle aspirate biopsy (FNAB) equipment may be the major explanation for the rise in PTMC detection (8). Traditional therapy for PTMCs has been surgical resection or thyroidectomy; however, active surveillance (AS) has emerged as an alternative strategy in the past three decades, with the goal of identifying the subset of individuals who would develop clinically and hence need rescue operations.

Active surveillance has been found to be a viable first-line therapy strategy for low-risk PTMC in recent trials (4, 5, 9, 10).

For low-risk PTMC, active monitoring was originally evaluated as a therapy option in Japan. Japan introduced active monitoring into standards in 2010, and the United States followed suit 5 years later in 2015. The Japan Association of Endocrine Surgeons (JAES) and the Japanese Society of Thyroid Surgeons (JSTS) issued the first guidelines for differentiated thyroid carcinomas, which included active monitoring as a therapy option for low-risk PTMC (11). Active monitoring as a therapeutic option for low risk PTMC was also included in the recommendations of the American Thyroid Association (ATA) in 2015. However, several clinical and technological aspects determine whether or not active monitoring is useful and feasible for low risk PTMC patients. For individuals with PTMC considered to be at “extremely low risk,” active monitoring is suggested as a treatment option according to the ATA's 2015 recommendations (12). Active surveillance can partially reduce unnecessary surgeries for patients diagnosed with PTCM, which is usually indolent and has a good prognosis, so that the possibility of over-treatment could be reduced in some patients.

However, occult lymph node metastasis (LNM) is still a possibility for clinical node-negative (CN0) PTMC. Therefore, the necessity and extent of surgical treatment option for low PTMC patients remain a controversial subject (6). The purpose of this retrospective study is to better understand the benefits and drawbacks of using preoperative ultrasound for the prediction of central lymph node metastasis (CLNM) in the cervical region, as well as to analyze data on occult central lymph node metastasis to determine the optimal mix of surgery, active surveillance, and avoidance of treatment in patients with PTMC. Examining the potential of an active monitoring method to prevent over-treatment in patients is central to the goals of this research, which aims to investigate the predictive aspects of CLNM of PTMC.

## Methods

This was a single-center retrospective study conducted on the data and medical records of the patients who were diagnosed with PTMC and underwent surgery at the Baotou Cancer Hospital, China, between January 1, 2018, and December 31, 2019. Data and medical records of the patients with PTMC who underwent monitoring and therapeutic procedures at the otolaryngology (ear, nose, and throat) division of Baotou Cancer Hospital, China between January 1, 2018, and December 31, 2019, were analyzed retrospectively for this research. The patients were all candidates for thyroid surgery, either lobectomy or complete thyroid removal. Prophylactic central lymph node dissection (CLND) was also done on the same side as the lesion, as recommended by the Chinese guidelines for the diagnosis and treatment of thyroid nodule and differentiated thyroid carcinoma. Comparisons and analyses were conducted on the preoperative ultrasound characteristics of PTMC, the pathological results post-operation, and lymph node metastasis.

SPSS (IBM SPSS Inc., Chicago, IL, Windows version 22.0.0) was used to conduct statistical analyses on the study's data, including the *t*-test, Pearson's Chi-square test or Fisher's exact test, and ROC curve and logistic regression. The normality of the data that was continuously collected was tested. Normally distributed research data were reported as means standard deviations (SD), whereas skewed variables were shown as medians (Inter Quartile Range). In order to compare and contrast the various groups, we utilized the *t*-test for normally distributed data, the non-parametric test (*Mann-Whitney U*) for data with a skewed distribution, and the Pearson Chi-square test for data based on counts. In the case of normally distributed data, Pearson's correlation coefficient was used to examine the intercorrelations between the variables, whereas in the case of skewed data, *Spearman's* correlation coefficient was used.

## Results

After applying inclusion and exclusion criteria, of 1,586 patients hospitalized for thyroid disease, 172 PTMC patients were included in this study [age: 48.32 ± 10.59 (SD) years old, and male/female ratio 31/142]. Ninety-eight cases of suspected lymph nodes were not detected *via* preoperative US, 20 cases (20.4%) of CLNM were confirmed *via* pathology, and 74 cases (43.2%) were found to have suspicious LN *via* preoperative US (Table 1).

**Table 1:** Clinical characteristics and results of US imaging and pathologic assessments of the patients.

**Category**	***N* (%)**	**US suspicious CLN**
All	172	74 (43.0%)
**Sex**
Female	142 (82.0%)	56 (75.7)
Male	31 (18.0%)	18 (24.3)
Age (mean ± SD) (year)	48.32 ± 10.59	47.62 ± 12.06
**Age group**
Group 1 age <45	57 (33.1)	26 (35.1)
Group 2 45 ≥ age > 55	63 (36.6)	28 (37.8)
Group 3 age ≥ 55	52 (30.2)	20 (27.0)
**Preoperative US TI-RADS grade**
3	10 (5.8%)	3 (4.1)
4a	42 (24.4%)	18 (24.3)
4b	77 (44.8%)	33 (44.6)
4c	1 (0.6%)	0
5	41 (23.8%)	19 (25.7)
6	1 (0.6%)	1 (1.4)
**Tumor site**
Unifocal	121 (70.3%)	49 (66.2)
Double or multifocal	51 (29.7%)	25 (33.8)
The max diameter (mean ± SD)	5.68 ± 2.48 mm	5.95 ± 2.32 mm
US capsular invasion	50 (29.1%)	31 (41.9)
US microcalcification	101 (58.7%)	48 (64.9)
**Postoperative pathologic results**
Unifocal	121 (70.3)	49 (66.2)
Double or multifocal	51 (29.7)	25 (33.8)
Capsule invasion	74 (43.0)	31 (41.9)
Nerve invasion	5 (2.9)	4 (5.4)
Fewer than 5 CLNM	164 (95.3)	–
Co-exist thyroid goiter	73 (42.4)	23 (31.1)
Co-exist Hashimoto thyroiditis	55 (32.0)	28 (37.8)
**Complications**
Recurrent nerve palsy	0	
Temporary hypoparathyroidism	9 (5.2)	
Permanent hypoparathyroidism	2 (1.2)	

Patients with lymph node metastases were found to be less common in the Group 2 (45 ≤ age <55 years old), compared to the Group 1 (<45 years old) and the Group 3 (≥55 years old) (*P* = 0.018). No statistically significant differences were found between any of the groups (**Table 3**). Table 2 displays postoperative pathology results.

**Table 2:** Statistical data of the patients with central cervical lymph nodes metastasis (CLNM).

	**Group 1 Age <45**	**Group 2** **45 ≤ age <55**	**Group 3 Age ≥ 55**	**Total**
*n* (%)	57 (33.1)	63 (36.6)	52 (30.2)	172
CLNM patients (%)	24 (42.1)	14 (26.9)	14 (26.9)	52
CLNM lymph nodes (%)	85 (64.4)	24 (18.2)	23 (17.4)	132
CLN dissected (%)	322 (35.2)	343 (37.4)	251 (27.4)	916

Using US imaging to identify CLNM has a sensitivity of 0.62, specificity of 0.35, and an area under the curve (AUC) of 0.63. For PTMC patients, the probability of central lymph node metastasis was highest among those who were male, over the age of 45, and whose tumors lacked micro-calcification on US imaging. The remaining parameters did not show any statistically significant link (Table 3).

**Table 3:** Statistical data of the logistics regression analysis.

**Category**	**OR (95% CI)**	***P*-value**
**Gender**
Male	1.00	
Female	2.09 (0.18–0.96)	*P* = 0.040
**Age (year)**
Age <45	1.00	
Age ≥ 45	2.09 (1.01–4.30)	*P* = 0.046
**US microcalcification**
None	1.00	
Sandy	0.48 (0.23–0.99)	*P* = 0.046

According to active surveillance exclusion criteria as per reported by Ito et al. (5) postoperative pathology confirmed 58 cases (33.7%) that showed the following characteristics: no metastasis near the trachea, located at the dorsal side of thyroid gland, no lymph node metastasis in six areas, no capsule invasion, and no high-grade malignancy.

## Discussion

The over-treatment of indolent lesions with mostly low malignant potential is not uncommon, but it may be caused by the lack of evidence and comprehensive understanding regarding the disease at that time. The progression of diagnosis technology has caused a rapid increase in PTMC cases. However, lack of accurate and precise classification and prediction still exists for the disease, which may increase the amount of unnecessary invasive treatments.

AS partially slowed down the PTMC over-treatment trend safely and prudently. Emerging evidences from active surveillance studies have demonstrated that many low-risk PTMC patients can benefit from active surveillance and their morbidity probability is reduced. This is caused by operations and follow-up radioiodine therapies with increased risk stratification *via* confirmed lymph node metastasis, including hypothyroidism, hypocalcemia, recurrent laryngeal nerve palsy, inconvenience and side effects of postoperative TSH treatment, salivary gland reaction, secondary leukemia, and other damage possibilities. High-risk patients were not considered for active surveillance by Ito et al. because of unfavorable lesion characteristics such as proximity to the trachea or dorsal thyroid surface, potential invasion of the recurrent laryngeal nerve, clinically apparent nodal metastasis, or high-grade malignancy on FNAB findings (5). In addition, the American Thyroid Association Guidelines outline a plethora of variables that classify individuals into high- or low-risk profiles, although these are generally pathologic abnormalities discovered only after surgical resection (13). Therefore, applying risk stratification pre-operation in clinical practice is difficult.

It remains unknown whether or not PTMC is associated with occult central lymph node metastasis, or whether or not risk stratification is upgraded. Moreover, It remains uncertain whether or not it is safe and controllable to wait until these hidden lymph nodes can be detected clinically. Likewise, it is unknown whether or not this will increase the local recurrence rate or the probability of secondary operations and side injuries. Lastly, how to accurately select these patients and conduct more detailed risk stratifications remains unknown.

PTC has a relatively high rate of lymph node metastasis so that lymph node metastasis derived from PTC first involves the central compartment (14). A meta-analysis (19 studies comprising 4,014 patients) found that the rate of central cervical lymph node metastases (CLNM) of PTC was 48.0% and the rate of lateral CLNM of PTC was 59.2% (15). Another meta-analysis found that, even without clinically cervical lymph node metastasis (cN0) PTMC, there was still about 33% (95% CI 29–37) of CLNM (16). Our finding was consistent with this study where we found that the CLNM rate of PTMC was 30.2%. A meta-analysis study reported that in PTC patients lymph nodes were involved in 80% of recurrences (17).

However, there is a lack of simple and reliable methods for conducting accurate preoperative judgments of occult lymph node metastasis and the biological characteristics of PTMC. Ultrasound accuracy when diagnosing central lymph nodes remains low. A meta-analysis concluded that for detecting central CLNM with ultrasound the pooled sensitivity was 0.33 [95% confidence interval (95% CI): 0.31–0.35], the specificity was 0.93 (95% CI: 0.92–0.94), the DOR was 5.63 (95% CI: 3.50–9.04), and the area under curve (AUC) was 0.69. In this study, we used pre-operative US imaging for detection of CLNM and the sensitivity was 0.62, the specificity was 0.35, and the area under curve (AUC) was 0.63. If the US imaging does not find a suspicious lymph node, then the lymph node FNAB target cannot be determined. There is not enough evidence to show that such PTMCs with CLNM are indolent and could be safely monitored. Moreover, this must be conducted preoperatively or at the latest intraoperatively to evaluate the central compartment LN status precisely. However, this study found that even if suspected lymph nodes were noted, it is hard identifying the suspicious LN found preoperatively *via* US was precisely consistent with the metastasis LN confirmed by the pathology postoperatively. Patients in this region have a lot of difficulties receiving preoperative molecular or gene detection or PET/CT due to economic limitations and medical insurance clauses. In turn, providing accurate information regarding central lymph nodes pre-operation is very difficult.

Not all patients are comfortable with observational treatment when presented with a cancer diagnosis, and many refuse active surveillance as a treatment option (4). Additionally, follow-up examinations are costly, time-consuming, and inconvenient for both patients and clinicians, which prevents utilizing them widely for detecting LN metastasis (18). In the outpatient department used by the authors, around less than 1/4 of PTMC patients who meet the standard willingly choose AS. However, most of the other patients, although they have been informed of the relevant details, choose surgical treatment for fear of cancer, and they have a strong desire for a radical cure rather than any kind of secondary surgery for recurrence. Therefore, it remains uncertain whether or not active surveillance risk stratification should supply psychological evaluation considerations. In this study, 33.7% of patients may be more suitable to join the active surveillance cohort. This is the first research to our knowledge to examine PTMC and overtreatment at a single location using preoperative ultrasonography and postoperative histology.

Lymph node metastasis (LNM) is a major recurrence predictor and affects PTMC patients' survival rate, although it does not seem to alter PTMC patients' 10-year disease-free survival rate (7). Updated technology in the future might be relied on to provide more objective and accurate primary lesions and lymph nodes. It can be predicted that technological progress will allow for more and more suspected metastatic lymph nodes to be found pre-operation. Therefore, it remains uncertain as to whether or not active surveillance can still be used for low-risk PTMC patients even if they have lymph node metastasis. Likewise, methods for stratifying the risk of lymph nodes remain unknown. More evidence may be provided by a large amount of data and long-term prognosis observations.

This study had some limitations that should be considered in generalizing the findings into the general population. The study's single-center retrospective design was a weakness, and even after removing potentially relevant data, it remained gaps that may introduce bias. There is not a single accepted method for quantitatively assessing mental health. Long-term surveillance and follow-up of patients in this research is still necessary to determine their prognosis.

## Conclusions

Despite a meteoric rise in the number of cases identified, mortality from intrathyroidal papillary microcarcinomas has been about the same. Active monitoring has arisen as an alternative to surgical resection with the purpose of identifying the subset of individuals who will develop clinically and would benefit from rescue operations. These tumors (particularly those between 1 and 2 cm) show no development during follow-up, grow at very slow rates, and can even shrink in size. Since papillary microcarcinomas are so common and observational results are so good, active monitoring may be a viable option for carefully chosen individuals. Active surveillance might benefit many PTMC patients and reduce damages caused by surgical therapies and related treatments, but PTMC treatments should also focus on the existence of occult lymph node metastasis, especially in patients with over 45 years old, male, tumor without micro-calcification in US. While accuracy of preoperative US imaging in the prediction of lymph nodes in the central cervical regions needs improvement, CLNM potential should be kept track of vigilantly in PTMC patients with microcalcification or suspected lymph nodes. Further studies should be conducted to improve the PTMC risk stratification accuracy. In this study, about 30% of the patients with PTMC had no active surveillance high-risk factors but required surgical treatment. Although informed of the details, PTMC patients' fear of cancer is still the main reason for choosing surgical treatment for active surveillance.

## Data availability statement

The original contributions presented in the study are included in the article/supplementary material, further inquiries can be directed to the corresponding authors.

## Ethics statement

The studies involving human participants were reviewed and approved by the Baotou Cancer Hospital, China. The patients/participants provided their written informed consent to participate in this study.

## Author contributions

BH, JW, and JF contributed to conception and design of the study. SH organized the database. ZH performed the statistical analysis. BH wrote the first draft of the manuscript. SH, JW, JF, and ZH wrote sections of the manuscript. All authors contributed to manuscript revision, read, and approved the submitted version.

## Conflict of interest

The authors declare that the research was conducted in the absence of any commercial or financial relationships that could be construed as a potential conflict of interest.

## Publisher's note

All claims expressed in this article are solely those of the authors and do not necessarily represent those of their affiliated organizations, or those of the publisher, the editors and the reviewers. Any product that may be evaluated in this article, or claim that may be made by its manufacturer, is not guaranteed or endorsed by the publisher.
